# Jejunal Intussusception: A Rare Adult Presentation of Lymphoid Hyperplasia

**DOI:** 10.1155/2019/9017863

**Published:** 2019-04-04

**Authors:** Linda Laham, Ratul Bhattacharyya, Manrique Guerrero, Jafar Haghshenas, Mark Ingram

**Affiliations:** St. Joseph's University Medical Center, Dept of Surgery, 703 Main St. Paterson, NJ 07503, USA

## Abstract

A 21-year-old African-American male presented to the emergency room with a sudden diffuse onset abdominal pain of one day duration. CT findings revealed mild telescoping of loops of small bowel and mesenteric fat in the left mid abdomen with no apparent masses. The patient underwent an exploratory laparoscopy revealing intussusception of the mid jejunum. As a fair amount of distention compromised safe navigation of the bowel, laparoscopic resection was not warranted at this time. Open approach allowed for segmental resection of the affected segment of the small bowel. This was followed by primary anastomosis. Pathological findings revealed focal reactive lymphoid hyperplasia with marked congestion in the lamina propria of the jejunum. The patient had an unremarkable postoperative period and recovered with no further complications.

## 1. Introduction

Intussusception is a potentially life-threatening condition involving intermittent crampy abdominal pain, occurrence of mucoid hematochezia, and threatened ischemia of the small intestine. Intussusceptions originate from telescoping of a segment of the gastrointestinal tracts into an adjacent section of the bowel [1]. The clinical presentation of this pathology classically involves abdominal pain or vomiting, as well as typical signs of an abdominal mass or rectal bleeding [2]. Compromise of mesenteric vessels, venous compression, and outpouring of mucus allow for these typical symptoms and presentation of a red currant jelly stool [3]. The incidence of intussusception is 1.5-4 cases per 1000 live births, with a predominant 3 : 2 male-to-female ratio [3]. Intussusception typically presents in infants 6-36 months of age and is a frequent cause of intestinal obstruction [3]. When in adults, intussusception is typically led by a pathological lead point of a neoplasm, adhesions of the small bowel, or resultant of weight loss surgery [4]. All together this phenomenon presents in less than 5% of all bowel obstruction cases in the adult population [5]. We present a rare case of a 21-year-old patient presenting with intussusception due to lymphoid hyperplasia.

## 2. Case Presentation

**Figure 1:**
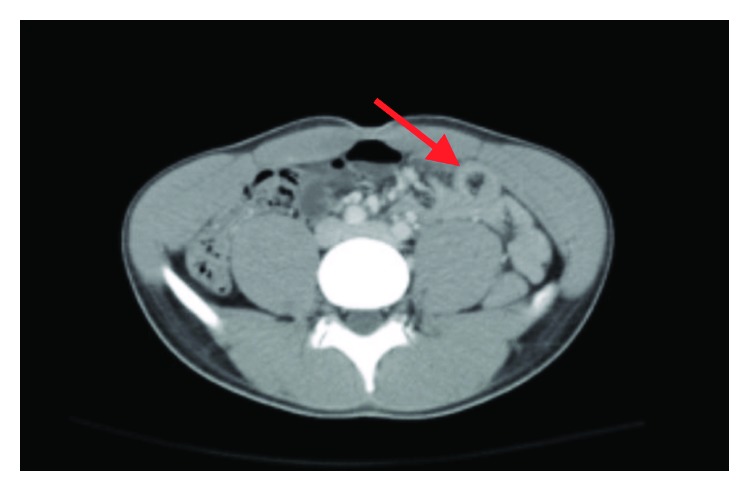
CT of the abdomen and pelvis shows bowel-within-bowel configuration (arrow), where the layers of the bowel are duplicated forming concentric rings.

A 21-year-old African-American male presented to our emergency department complaining of sudden onset of diffuse abdominal pain. His history was significant for recurrent episodes of gonococcal urethritis and no other ailments. He described this pain as diffuse and constant pressure that started suddenly that morning and had progressed throughout the day. Patient was hemodynamically stable with leukocytosis at 11,200 and a positive urinalysis. Computed Tomography (CT) revealed mild telescoping of loops of the small bowel and mesenteric fat in the left mid abdomen (Figure 1). No obvious bowel obstruction or definitive masses were seen on imaging.

**Figure 2:**
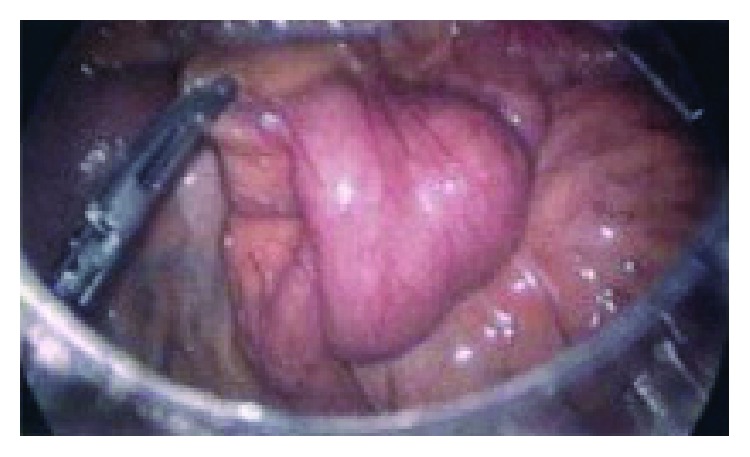
Invaginated loop of the jejunum on laparoscopic exploration preresection.

Persistent abdominal pain after 24 hours of observation prompted diagnostic laparoscopy revealing intussusception of the mid jejunum. This prompted open exploration, segmental resection, and primary anastomosis of the jejunum. Pathology reported marked congestion and focal reactive lymphoid hyperplasia in the lamina propria of the invaginated bowel. The patient was discharged home postop day 2 with an unremarkable follow-up. CT findings revealed mild telescoping of loops of small bowel and mesenteric fat in the left mid abdomen. Uncertain by these radiographic findings, exploratory laparoscopy was initiated, profoundly confirming inflammation and telescoping of the jejunum (Figure 2).

## 3. Differential Diagnosis

A patient presenting with diffuse and vague abdominal pain may have a small bowel obstruction, appendicitis, perforated viscus, inflammatory bowel disease, or other causes of peritoneal inflammation [4]. Noninflammatory causes can also include such etiology as Meckel's diverticulum and other obstructing lesions. Such pathologies as polypoid lesions and tumors of benign and malignant origin must also be considered with a high index of suspicion [4].

Due to the lack of previous surgical history, suspicion for intraperitoneal adhesions was low. A small bowel obstruction of unknown cause ranked amongst the top of our differentials. CT was important in investigating the etiology of the continuous abdominal pain to avoid missing catastrophic sequelae. Laparoscopy and ultimately an open laparotomy were deemed imminent in defining our root cause. With no knowledge of abdominal adhesion or history of hernias, our scope of differentials has to exceed more than just a small bowel obstruction.

## 4. Treatment

Contrast air enema reduction was bypassed due to indication for surgical resection as our patient was of adult age and at risk for malignancies and recurrence [5]. This approach would prove to be diagnostic and therapeutic.

## 5. Outcome and Follow-Up

Following the segmental resection and primary anastomosis of the proximal bowel, our patient had no significant postoperative complications and was discharged on day 2 after the procedure. The patient had an unremarkable follow-up.

## 6. Discussion

We present a rare case of jejunal intussusception causing bowel obstruction and ischemia in an adult patient. Acute presentation of this disease is usually limited to infants of less than 1 year of age and affiliated with such complaints as red mucinous stools [5]. Yet, we present a young adult patient with nonspecific complaints leading to an extensive course in diagnosis. CT imaging was inconclusive in our diagnosis, thus turning to laparoscopy; we were able to proceed with laparotomy and segmental resection as a definitive treatment for this patient.

Cases of intussusception in the adult population are often easily diagnosed via CT imaging and confined to typical etiologies such as adenomatous polyps, lipomas, Peutz-Jeghers syndrome, neurofibromas, and other benign and malignant tumorous diseases [5].

Ultrasound imaging has proven useful in such cases in the past. Yet sensitivity in this modality is limited to the experience of the technicians and radiologists involved [6]. Cases involving diagnosis with an ultrasound are also usually recommended for further confirmation with such modalities as CT [7].

Pathology from our case revealed lymphoid hyperplasia as the etiology in our lead point. Laparoscopy revealed a significant yet viable portion of the invaginated jejunum approximately 4 feet from the ligament of Treitz. Reduction and further inspection of the involved bowel confirmed indication for segmental resection over en bloc resection. With no concerns of bowel ischemia at this time, we were able to proceed with a simple resection and anastomosis. Current modalities of practice support intraoperative decision towards definitive surgical treatment, depending on such variables as viability and risk of a short gut [8].

Lymphoid hyperplasia though common in the pediatric population, can also lead to acute abdominal pathology in the adult population. Though no presence of malignant disease was found in our patient, such etiologies as lymphoma would also be relevant to such a case [9]. Though extremely rare, such occurrences as Burkitt lymphoma have also been reported in adult intussusception [9]. In a patient of African origin, the chances are slightly higher [9]. We present a rare case of unresolved abdominal pain in an adult, due to jejunal intussusception requiring definitive treatment via segmental resection. In an adult patient, differentials of appendicitis, abdominal adhesions, enterocolitis, and ischemic bowel may be more likely in a patient with abdominal pain described as diffuse and unresolving [8].

We suggest intussusception as a worthy differential for adult patients presenting with obstructive symptoms of unknown origin and nonresolving abdominal pain. These cases should prompt aggressive surgical intervention as other methods can delay necessary diagnostic and therapeutic resolution.

## 7. Learning Points

The following are the learning points:

1. Intussusception as a worthy differential for adult patients presenting with obstructive symptoms of unknown origin
2. Lymphoid hyperplasia, though common in the pediatric population, can also lead to acute abdominal pathology in the adult population
3. Exploratory laparotomy and resection are best indicated to understand the underlying causes of adult intussusception
